# Address before the Virginia Society of Surgeon Dentists

**Published:** 1843-12

**Authors:** James D. McCabe


### ARTICLE IV.

ADDRESS *before the Virginia Society of Surgeon Dentists,* on the evening of their first Annual Meeting, Oct. 11, 1843. By JAMES D. McCABE, D. D. S.

[Published by request of the Society.]

*Gentlemen of the Virginia Society of Surgeon Dentists:—*

THE duty you have assigned me, to address, at its first annual meeting, the Virginia Society of Dentists, might have been confided to an abler exponent of your purposes, and the character of your cherished science, but I can confidently say, to no one who feels a deeper interest in your success, or who is more willing than your speaker to tax to the uttermost his abilities in support of your undertaking. Nearly twelve months of experience have passed, and I rejoice to see assembled around me to-day, the same faces that I saw at our institution, and others beside, all beaming with satisfaction, and noble determination, giving evidence of zeal, rekindled by the collision of mind, and emulation excited by mutual perseverance.

The present anniversary is full of rich auguries, and exhibits already the dawn of a brighter day to our profession, in this time-honored commonwealth; and though there may yet linger "some dregs of ancient night not quite purged off," along the orient of our hopes, yet may we confidently expect, that the persevering labors of the faithful and intelligent, will ultimately scatter the last cloud from the horizon, and shed a flood of healthful light upon the meridian of our success. It is at least a source of encouragement to those who have grasped the sickle of investigation and cheered their compatriots with the cry of improvement and reform, to know that in the field which spreads out before them, there are flowers of philosophy and rich fruits of wisdom, whitening to an abundant harvest, and promising amply to repay the faithful reaper in their fruitful fields.

That branch of science, gentlemen, in which it has been your pleasure to embark, and for the cultivation and improvement of which you are associated, is yet in its infancy, as a distinctly cultivated branch of the art of healing, though it ranks, in its origin and claims, with the oldest and most elevated divisions of its kindred branches. While surgery and medicine have arisen from their once humble vocation, and from the compounding of a few simples, and the setting of a fractured limb, became noble and expansive sciences, dentistry has been neglected, or else had its distinct character destroyed by being merged in the science of surgery, as part and parcel of its minor manipulations. Notwithstanding this has been so, it has its special claims to consideration, and a very few years have been sufficient to vindicate, in a good degree, its obscured claims, and to present it in its native excellence, associated with every other branch of the science of healing; reflecting, in some degree, the improvements of each, and partaking in the great principles of all.

Dentistry presents to the view of its scientific votary, an extensive prospect, every visible point of which, when gained, presents a still increasing horizon. True, it professes to treat of the diseases of the teeth and gums, yet the various affections, both of a local and constitutional character, to which their diseases give rise, demand an acquaintance with the laws of the human system, its ills and antidotes.

Comparative anatomy, with its ever varying and interesting comparisons and observations, presents an almost shoreless ocean, in which islands, aye continents, abound, never yet pressed by the foot of a discoverer, or seen through the glass of the explorer; upon this ocean, the surgeon dentist may launch his bark in quest of scientific adventure, and spend a long life amid the ever increasing wonders that arise around him, in quick and varying succession, to reward his toil.

The learned labors of *Malpigi, Duverney, Laurance, Winslow, Ritzius, Bertin, Jourdain, Hunter, Bichat, Cuvier*, and others, present but the imposing outline of a map of discovery, to be filled up by the discoveries of others; and to no branch of the healing art does this most interesting science more directly address itself, than to dental surgery. Special anatomy and physiology, with their deeply interesting developments, constitute another field of study, which, in order, should properly have been first adverted to. The disposition and arrangement of the various parts of the human system, a knowledge of the various tissues and their functions, both in the normal and abnormal condition, is absolutely requisite to one whose attention is directed to the treatment and cure of diseases which materially affect the whole economy, by derangements of the nervous system, and through the agency of the digestive apparatus, and alimentary canal.

Therapeutics, as embracing materia medica and the practice of physic, has also its claims for general acquaintanceship, together with a knowledge of chemistry, both analytical and synthetical. Another constituent in the character of a *well educated dentist,* is an acquaintance with practical surgery, and of consequence a good degree of tact in mechanics, in order that he may understand the treatment of existing disease, and be prepared, in a skilful manner, to remedy the loss of such valuable organs as the teeth, by supplying artificial substitutes.

You will perceive, gentlemen, by this very cursory view, (do I place the standard too high? I think not,) that the profession of dentistry is not what a great many have supposed it to be, that of (to use the language of a wit) "a mere mouth carpenter and tooth joiner;" or what many who have called themselves by its name have made it, but a branch of that noblest of all sciences, which has for the subject of its investigations, MAN, in all his relations and conditions in life and in death. That I have not estimated the qualifications for practice too high, is most obvious when we consider that the best performed operations in dentistry, where mere mechanical skill is concerned, will, under certain conditions of the system, prove decidedly injurious. *Let me not, however, be misunderstood, or for a moment considered guilty of the glaring inconsistency of asserting that, in the common use of that term, the most finished medical education will qualify a man for the practice of dentistry!* There must be superadded that particular study, and artistical tact, which specially belongs to our art, without which no man should dare assume its responsibilities. It is a most notorious fact, and from it our art has deeply suffered, that some of our most distinguished medical men are grossly ignorant of the first principles of scientific and skilful dental practice.

It is true, the dental practitioner is not called to tread the loftiest path of medical responsibility. It is not his vocation to keep sad vigil beside the couch of death; to count the ebbing strokes of life's failing tide; to utter the fearful words, which, like ice-bolts, rive the heart; or speak the blessed accents of encouragement and hope, that clothe the pale countenance of the midnight watcher with smiles; yet, in the sphere of his duty, the responsibility is immense, his malpractice may prove the proximate cause of maladies, the responsibility in the treatment of which, the divisions of science compel another to assume.

Viewing dentistry, therefore, as an important branch of science, we may well be astonished at the abuses and charlatanism with which it has abounded, and the apparent neglect with which it was for a long time treated. When surgery was released from the hands of the ignorant barber, and elevated to its true position, it seemed by general consent conceded, that he whose high function it was to remove the superfluous hair from the face and poll the head, was well qualified to take under his protection the teeth, and though a series of efforts were made to show the importance of elementary knowledge, and proper skill in the management of these delicate organs, by such men as *John Hunter, Sydenham, Astley Cooper, Bell, de Blainville, Richerand, Rush, Fox, Goode, Hooper*, and other writers of distinction, still was the subject regarded as comparatively unimportant by the mass of medical men, and left to struggle for existence, if not entirely in the care of barbers, in the hands of those equally barbarous. Some skill in the mechanical use of tools, and in introducing metallic filings into the cavities of carious teeth, was considered by medical men themselves, sufficient to qualify one to enter upon the treatment of disease in organs among the most useful and delicate in the human system. The result was, *plenty of work for the doctors*, in broken constitutions, and a long catalogue of nervous affections. Even to the present day, the text-books in anatomy and physiology, used in our medical institutions, contain gross errors, founded upon long exploded doctrines with relation to the teeth, a circumstance for which we cannot furnish a rational solution, unless it be found in the comparative indifference with which the subject is regarded by most of the exclusively medical writers.

These facts furnish grave and sufficient cause, if there were none other, why dentistry should be zealously cultivated as a distinct branch of the healing art, and why those who appreciate its dignity and importance, under the present state of affairs, should associate, as you have done, to develope its resources and establish its well ascertained principles of practice for the mitigation and removal of those complicate and distressing affections, which destroy the health, and mar the beauty of the human face divine. Dr. James Johnson, of the "Medico Chirurgical Review," writing on this subject, very justly remarks, "there appears to be more valid reasons for dental surgery furnishing a sort of separate art, than for the surgery of the eyes and the ears being made so.

In the former, much mechanical contrivance is requisite, so much so as to render it exceedingly improbable that any but a special artist could excel in it; but the operations for the eyes and ears can be as well performed by a dexterous surgeon as by an oculist or an aurist." And I may add to this expression of opinion, that until dentistry was cultivated with the energy and zeal demanded by a "separate art," the most wild and ridiculous vagaries were indulged in by eminent men, with regard to the teeth.

Aristotle, that universal genius of antiquity, declares most positively, that men have more teeth than women, and, that this is the case with several other animals. This furnishes an evidence of the inexact manner of the philosophy of former times, leading men to assert, *ex cathedra,* as fact, what the slightest examination of every recurring fact would have convinced them was no fact at all. Perhaps, however, this philosopher thought with Homer, that the teeth were "barriers opposed by nature to slips of the tongue and the abuse of speech," and, as the ladies indulge more freely in the exercise of the unruly member, he therefore concluded that nature had furnished fewer barriers to its exercise.

*Pliny*, who doubtless speaks the opinions of the scientific of his day, assures us, that "the human teeth contain a *poisonous virus,* and that a bite from them will destroy weak animals."

*Thomas Bartholen*, of a more modern age, and who ranked high as anatomical authority, declares that he saw a man in whose jaw grew an *iron tooth,* and he actually enters into an argument to prove that it is reasonable. This is but a very brief specimen of the wholesale absurdity, which, in every age, distinguished writers upon the teeth, from Hippocrates to Blake; and, in some instances, even since the latter period, referred to. We learn from undoubted sources, that the teeth attracted attention at a very early period of time; and that they were classed in their treatment, as a separate division of the healing art, from medicine and surgery, is evidenced by the declarations of the most distinguished Greek, Roman and Arabic writers. When ancient Memphis reflected the glories of the Pharaohs, and Thebes, which even now in ruin arrests the admiration of the traveller, was still in its infancy, when its hundred gates poured its tides of population out, and its marts echoed to the hum of industry and trade,—when the mountain tombs of its kings were tenantless, and its young glory undimmed, our art was cultivated ; and, with its kindred sciences united in conferring benefit upon man, and by attracting attention to its demands, laid the foundation of its future usefulness. Among the nations which succeeded to the scientific glory of Egypt, it continued to be noticed, though not always with equal complacency with its kindred branches. Through the long night of barbarism and ignorance, in which the Latin empire expired, and until the revival of letters in the west of Europe, it found a home, doubtless with science, in the cloistered home of the cowled priest, and was by him practiced in its simpler operations as a minor part of surgery, until the council of *Tours*, in 1163, issued its decree, forbidding the shedding of blood by the ministers of religion, by which the priests were compelled to desist from any pursuit involving loss of blood to their patients. "*Ecclesia abhorret a sanguine*," "the church holds the shedding of blood in abhorrence," was the language of the council, and the abandonment of surgery by the monks removed the only barrier to the preferment of attendants on baths—barbers—mountebanks and ignorant itinerants; and surgery, which at that day consisted almost entirely in blood letting and tooth drawing, became, in a special manner, the pursuit of the barber; this class of persons were legally incorporated in several countries of Europe, under the designation of "*barber surgeons.*" In England, especially, they continued their vocation until the reign of George III., when they were suppressed by legal enactment. In some countries, to this day, they continue their pursuits.

Within the last fifty years, dentistry has been cultivated with more ardor, and determination ; and it is only within that time that it may be said to have assumed any thing like a respectable standing, either in England or on the continent.

To the labors of the indefatigable Blake, we may mainly attribute the increased importance of the science, and while we may be called upon to divide the laurel between him, Fox, and John Hunter, the largest portion of that wreath, in this department of practice, must forever adorn the modest brow of him, who labored in performing the manipulations for which his genius furnished the rules. I would not dare to essay to pluck one gem from the crown of the immortal Hunter, he stands solitary and alone in his glory, the greatest mind of his age, like some lone brilliant star in the mid heavens, lighting up with its beams the surrounding gloom. Uneducated, and without the previous preparation so important to secure success, he grasped the torch of investigation with untrembling hand, and boldly led the way through the mazy labyrinth of science, and in the memory of his almost incalculable achievements, *has* left behind him an incentive and example to the determined wooer of knowledge, of what may be accomplished by industry and perseverance, demonstrating in his whole life, the assured truth, that wisdom has inscribed on the door of her temple the universal invitation, *enter!*

Much, however, as we may justly owe to the writers and practitioners of the old world, it was nevertheless reserved for the profession in the United States to give an elevation and character to dental surgery, to which it had not previously attained, which in its progressive developments, is destined to give it *caste* with the most perfectly cultivated sciences. The very abuses to which it was exposed for years, in this country, tended to effect the very improvements of which we boast, and introduced the instrumentalities for its permanent benefit.

The parent of all these abuses was the easy access to the profession, occasioned by the small importance attached to its manipulations. The surgeon and physician, united with the public, in declaring, that a small degree of mechanical dexterity was alone demanded in the performance of the necessary operations indicated by the disease of the teeth, and this notion, based upon the supremely ridiculous doctrines which so extensively prevailed at one time, teaching the almost entire disconnection of these organs from the dependencies and sympathies of the general system, induced countless numbers to embark in the profession of dentistry, who, in the commencement of their professional career, were scarcely equal to the duties demanded by the erroneous opinions entertained of the necessary qualifications. But, among these numbers, there were some master minds, who actuated by a noble ambition, and emulous of distinction in their calling, gave a new impulse by their industry to the neglected science. These persons would never, doubtless, have dreamed of engaging in dentistry, but for the low estimate of its requirements which then extensively obtained, yet having engaged in it, they brought all the power of strong minds, and persevering industry to the investigation of its principles, and by their unwearied diligence, became the conservators of a noble science, and the benefactors of the human race.

We cannot regard the giant strides with which our art has marched on to eminence in the last few years, without experiencing feelings of the most lively gratitude to those, who, with minds well disciplined for the work, have stood in the front rank of the profession, raised its soiled and degraded banner from the dust, and given its ample folds to the breeze, with the cheering cry of—onward!

The genius of science has enrolled their names upon her imperishable tablets, and has awarded them a niche in the pantheon of her glory, alongside the noble spirits of the past.

I trust I may not be deemed invidious, or charged with an attempt to detract from the just merit of others, if I follow alike the dictates of friendship and of justice, in attributing many of the successful efforts to elevate our profession, in the United States, to the indefatigable labors of Dr. C. A. Harris of Baltimore, and his co-laborers, Drs. Hayden, Brown, and Parmley. Of Dr. Harris I may speak from the knowledge of years, derived from the closest and warmest personal and professional friendship. I *know* his unwearied labor for the advancement of his profession in respectability and standing—his industry as a writer, often at great sacrifice of ease and interest—his ardent efforts for the institution of instrumentalities and facilities for the cultivation of *"the dental art,"* such as has been furnished by the American Society, and Baltimore College. Whatever others may have done, and they have done much, very much to advance this cause, Dr. Harris must be regarded as the master-spirit of the enterprize, the devoted, the ardent friend of the profession. An accomplished practitioner, urbane and gentlemanly in his deportment, he occupies a position in the college he assisted in founding, which the interests of the science demands, and to which his own modest worth entitles him. I hope this digression, if it is so regarded, will be pardoned, in view of the justice attempted to be done to those to whom we owe so much as a profession.

The establishment of the American Society of Surgeon Dentists, presented a new era in the history of the profession; attracted by its utility, the extremes of the country have been united, and a mass of intellect and professional talent brought together in contact, which by collision, is like the smitten steel, annually scattering its scintillations of wisdom through the length and breadth of the land. Its published transactions present a voluminous exhibition of skill, and professional observation, and is full of bright auguries of the future. Already, in the few short years of its existence, has it raised the standard of dental requirement and professional education, and this effort, aided as it now is, by the establishment of the Baltimore College, in its healthful effects upon the future, will bring a bright day to dentistry in the United States. Already has this effort been felt across the Atlantic, (and to the honor of America be it said,) in the publication of a dental journal, and a determined effort to form a national society of dentists in the metropolis of the British empire, thus presenting two great nations engaged with generous emulation in the cause of science.

The formation of the Virginia Society, adds another link to the chain of improvement and reform, and as the devise of the seal you have selected indicates, the sun has at length arisen upon the old dominion, scattering the gloom of ignorance—the night of quackery and empiricism.

The great question for this society to determine, so far as its relations with the public are concerned, is the advantage, mutual and reciprocal, to be derived from its formation. Without being too prolix, it would be impossible on this occasion to do justice to this question, a very cursory view of its solution must suffice.

The first great advantage to be derived from its formation will be found in the union effected among the members of the profession, and the personal contact into which they will be brought, for we should be doing injustice to its honorable and scientific members to suppose that any *one of them,* will withhold his influence from the enterprize. The acquaintanceship thus formed, and the mutual consultations to which it will give rise, while they serve to elicit the result of much experience and observation, will, in their obvious tendencies, awaken an *esprit du corps,* and establish an united effort for the highest grade of professional education allowed by our art. I think that I may hazard the assertion, that there are no body of men in our state who can lay claim to higher attainments, or more thorough qualification for the pursuits in which they are engaged, than the *majority* of dentists; if, therefore, we can bring these scattered parts together, and unite them into a well organized body, the sole object of which is to elevate the science, and diffuse intelligence; our subsequent labor will chiefly consist in giving proper direction to our efforts, and we shall have laid the foundation of permanent usefulness.

It seems to comport with the genius of our free institutions, that great public benefits are most effectually secured by associations for their prosecution; heresy in church and state, in science and letters, individuals of kindred pursuits, are wont to unite for the advancement of great enterprises, bringing the united energy of the many to accomplish what would of necessity fail, if solely dependent upon individual exertion.

In a profession like ours, as it has for years been constituted, the voice of the solitary reformer is lost amid the din of professional jealousies, and the pride of professional opinions. What uttered by one, would be regarded as heresy, or an attempt to obtain professional reputation, spoken by the many, assumes the tones of an oracular voice, and finds in the listeners, willing disciples to its teachings; this then is an advantage derived from association, and its name is *legion.*

There are many evils to be cured, and abuses to be corrected, before we can realize the full amount of good, attainable by our union, and these evils and abuses necessarily grow out of the want of a well regulated and sound system of professional education; to effect this desideratum, will constitute an important and indispensable first step in our operations. We must demonstrate to community, that dentistry is not what ignorance has too long regarded it to be, an unimportant branch of surgery, which can be safely confided to ignorant operators. That the dentist has to do with *living* organs of the human system, and that a knowledge of the structure and functions of that system must form the basis of all rational practice; to effect this, our annual transactions should present a volume of practical information, derived from the observation and experience of our members;—not only so, but the undivided influence of our union must be made a barrier to the entrance of any one into our profession who is not qualified for its duties, and who is not possessed, at least, of the elementary knowledge of science and letters, which will qualify him, by patient industry, to add to its respectability, and to increase, in the course of time, its stock of useful professional knowledge. In the discharge of this high duty we should know no man after the flesh; the interests of community are in some degree in our keeping, and while we are conscious of the responsibility, we should labor diligently to acquit us in the delicate and highly responsible trust. There is one important consideration, however, which merits our most serious attention; while quackery and empiricism should at all times be frowned down, and exposed, we should be exceedingly careful *not* to assail professional reputation, or discountenance our brethren, because of difference of opinion. It has been too common for persons calling themselves dentists, sadly to forget, that a prime constituent in that character, is gentlemanly feelings and deportment, or that slander, prompted by narrow feelings of professional jealousy, is a *moral felony*, which, in the estimation of all honorable minds, excludes its perpetrator from the association of the virtuous and the good. I have always found, and my experience is confirmed by the observation of others, that he who is willing to trifle with the reputation of another member of the same profession, or lessen him in the estimation of the public, is either an ignorant quack himself, or grossly deficient in the practice of common honesty. You will always find that a practitioner of any science, if by education qualified for his profession, and by habits of association and principle, a gentleman, is extremely loath to censure the practice of another, however much he may differ from that other, unless a sense of public justice demands it at his hands. Much will have been gained by our association, if we effect the removal of this grave evil. If we are called upon in the legitimate exercise of our professional duties to expose quackery, let us do so with firmness and decision, without ostentation or coarseness, recollecting that no act by another, however disgraceful, will ever justify in the exposure, the use of vulgarity or imperiousness, by which the dignity of character may be compromitted, and especially should we be so prepared to expose such things, as not to be guilty of the crime we charge upon others. There is another very important end to be obtained by our association, which will be secured by an union of action, to discountenance, that most disreputable, and to a sensitive mind, disgusting system of professional puffing, often resorted to by members of our profession, for the purpose of promoting selfish ends; the most gross and impious frauds are, by this means, practised upon the public credulity, by long and windy professional cards, supported by an array of names, as recommendations, calling attention to new materials for plugging, new modes of artificial insertion, patent instruments for the extraction of teeth, and divers other strange things known only to the advertiser. I have always regarded such cards as evidence of ignorance and charlatanism, and their perpetrators a disgrace to a noble art. Gentlemen of this feather are always ambitious of distinction, to which they seek to make their way by the unmeasured abuse of every body but themselves; *and if as is often the case, (as I am told it is,) they can purchase the honorary degree of M.D. for* THIRTY DOLLARS, *without being able to distinguish a tincture from a decoction, they parade it as a suffix to their names, and turn up their noses at those who are too honest to wear honors to which they feel themselves unentitled, or to assume names and titles that do not properly belong to them.* I was much amused by an anecdote related to me by a medical friend, in the lower country, of one of these mountebanks. He had extracted a superior molar tooth for a gentleman, who became much alarmed at an excessive hæmorrhage which supervened; after some hours had elapsed, he sent for *the dentist* to know what should be done to stop it; he very gravely informed him that it was necessary *to take up the bleeding artery*, which he proceeded to do, by passing a cotton thread through the edges of the gums and drawing them together; unfortunately for his reputation, the cure was not completed, the bleeding continued, and the physician who related the circumstance to me, was called in, who by removing the ligatures, and applying the compress, succeeded in "*the old fashioned*" way in arresting the hæmorrhage.

Against these things, it behooves the reputable members of the profession to record their unchangeable disapprobation, as I feel assured they have always done in their individual capacity, but much more should it now be done by the voice and sanction of their associated wisdom.

There are a number of other evils which our association will have a tendency to cure, and though we may be deprived of legislative aid, in the form of corporate existence, unitedly acting, we will suffer but little from its absence; the public will accord to us, all that the exceedingly wise law-makers have refused, and a consciousness of the rectitude of our actions will be our rich reward.

One great and absorbing object of our association, has been declared to be, the furnishing of a sound system of dental education, and to this we should most actively address our exertions. The literature of our profession is most rapidly and ably increasing, and in the United States, we can boast the best practical writers of the last century; it behooves us then, in the absence of a school of dentistry in our own state, to throw the weight of our whole influence in the scale of the Baltimore College, and at the same time to prescribe such text-books to those who intend presenting themselves before our board of examiners, as will enable the society to test most fully the qualifications of all upon whom its honors are conferred. Upon the proper management of this department of the society, will mainly depend its respectability and usefulness. While I would be the last person on earth to offer any inducement to a student to omit any advantage of which he can avail himself, in completing his education, but would urge every young man desirous to enter the profession, to avail himself of the advantages offered by THE ONLY DENTAL COLLEGE IN THE WORLD; I would nevertheless counsel an effective organization of that department of our society, that we may, in some good degree, subserve the interests of the profession, by furnishing the facilities for graduation to those who have more merit than means, to entitle them to the honors of our art.

You know, gentlemen, that at this day the obtainance of a degree, and the possession of a diploma, are thought to confer but little honor, from the facility with which they can be procured; it will, therefore, depend upon the *diligence* and *care* with which the examinations of this society are conducted, whether its parchments are considered more valuable. Let the profession, let the world know, that no candidates will be recommended by the committee, for the honors of the society, unless he is qualified, morally as well as professionally, for our association—that his character as well as his attainments will have to pass the tests of the severest scrutiny, before he receives our certificate of competency, and is sent forth as a qualified dentist. Let this course of policy be pursued, and maugre the acts of *pseudo* law makers, our dear old Virginia will have taken the front rank in the glorious efforts now making for the diffusion and maintenance of a sound system of scientific professional knowledge; upon the glittering spear she wields, shall perish the miserable charlatanism with which the art has been cursed, and her sons shall go forth from our halls, like *Pallas* from the brain of Jupiter, full armed for conflict with the hydra of dental disease.

To the young gentlemen who have presented themselves for examination, and who have passed most honorably the ordeal of your able committee, I may be permitted to say a few words of advice and congratulation.

The profession you have selected, and to which your attention has been closely given through the period of your students' probation, is highly honorable—none can present objects of more interest to engage the human mind, or afford a larger field of intellectual pursuit; it at the same time confers fearful responsibility. It would be unnecessary to say to you, fresh as you are from your text books, and familiar as your examination has proven you to be with the principles of the science, that vast and imminent consequences depend upon the skilful and faithful performance of your professional duties. In your intercourse with your fellow citizens, be known only as instruments of good, in removing pain and contributing to the preservation and health of the beautiful organs entrusted to your care. Recollect that you will never cease to be students, every day will add a new page to the volume of experience; patient observation, and the judicious classification of the facts from which you will deduce your practical rules, will demand at your hands, patient and unwearied industry; if, for a moment, you are disposed to relax your efforts, let a sense of self-respect, and professional dignity, connected with your responsibilities, re-nerve your minds and strengthen your resolves. Be actuated by a noble ambition to be first in your profession, if labor and study will make you so. This is the only, this the highest evidence we ask to satisfy us that you are worthy the distinction we have conferred, the confidence we have reposed. I congratulate you upon your preferment, and speak the feelings of this whole society, when I give you a most hearty welcome into the ranks of the profession.

Gentlemen of the Virginia Society, I have thus briefly and hurriedly presented the topics which seemed to me most proper for your first annual address. In doing so, I have studied great plainness, and, so far as I could, studiously avoided ornament; I have been too anxious that we should gather the *fruit* of our toils, to amuse you with the *flowers* that might have been gathered by the wayside, for our profession is not barren of themes to awaken the pride of oratory, and call forth the eloquence of song; yet must we essay to remove the rubbish which obscures these latent beauties, ere we wreathe our altars with roses, or challenge the song of the minstrel to celebrate its beauties. To rejoice in triumph, belongeth to him who putteth off his armor, not to him who has just put it on, and is about to go forth to battle. During the past year much cause of gratulation has been afforded you in the approbation of your friends, the friends of science; they look upon your labors with the deepest interest for your success; and I feel well assured that they will be gratified with the result. The germ you have planted, I doubt not, will become a noble tree, bearing healthful fruit for the healing of the nations. The handful of corn you have scattered upon the mountain-top will produce an abundant harvest of golden grain, the rich sheaves of which will be cut, bound, and garnered by generations yet to come.

Difficulties may lie before you, but they will be removed, while around your pathway, freed by your own hands from various weeds and poisonous reptiles, shall bloom in rich exuberance, flowers of usefulness, and the rich fruitage of success; while ye yourselves, engaged in doing good, and laboring to fill up the measure of your days in usefulness, will go down to your graves in peace, the smiles of God around you, and in your bosoms man's holiest, richest treasures, a peaceful conscience, and the hope of endless rest.

